# Supplementary material for: A Buried Water Network Modulates the Activity of the Escherichia coli Disulphide Catalyst DsbA
Source: Antioxidants (Basel). 2023 Feb 4;12(2):380. doi: 10.3390/antiox12020380 (PMC9952396; doi:10.3390/antiox12020380)
Supplement: Supplementary file 1 [file antioxidants-12-00380-s001.zip › antioxidants-2181489-supplementary.pdf]

Supplementary Information

A buried water network modulates the activity of the *Escherichia coli* disulfide catalyst DsbA

Geqing Wang,[1,*] Jilong Qin,[2] Anthony D. Verderosa,[2] Lilian Hor,[1] Carlos Santos-Martin,[1] Jason J. Paxman,[1] Jennifer L. Martin,[3] Makrina Totsika,[2] Begoña Heras[1,*]

[1] Department of Biochemistry and Chemistry, La Trobe Institute for Molecular Science, La Trobe University, Melbourne, Victoria, Australia
[2] Queensland University of Technology, Centre for Immunology and Infection Control, School of Biomedical Sciences, Brisbane, Queensland, Australia
[3] Griffith Institute for Drug Discovery, Griffith University, Nathan, Queensland, Australia

To whom correspondence should be addressed: Prof. Begoña Heras (b.heras@latrobe.edu.au) and Dr. Geqing Wang (g.wang@latrobe.edu.au)

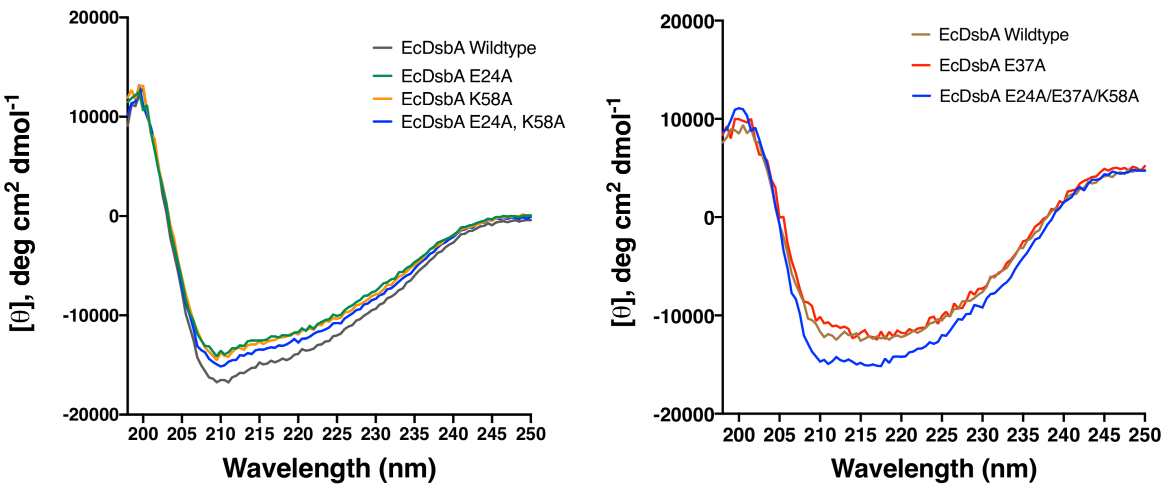

Figure S1. CD spectra of EcDsbA wildtype and mutants. Wavelength spectra were collected between 190 and 250 nm for DsbA mutants at 0.2 mg/ml, in a buffer of 100 mM sodium phosphate, pH 7.0, 50 mM NaCl, 1 mM EDTA using 1 mm quartz cuvettes with a step size of 1 nm. CD spectroscopy wavelength scan results showed that the overall fold and distribution of secondary structural elements were not affected by the introduced mutations.

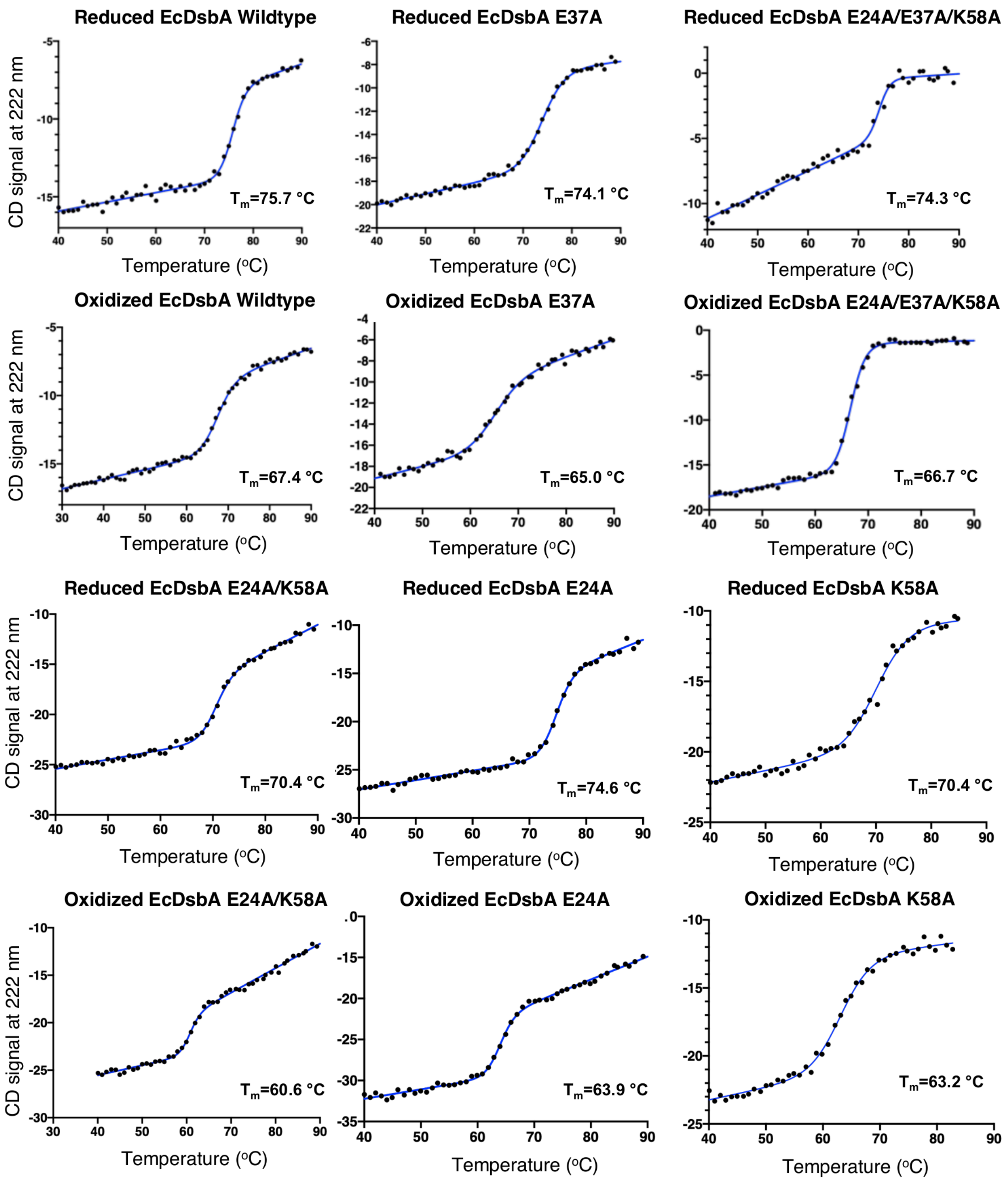

Figure S2. Thermal unfolding of EcDsbA wildtype and mutants monitored by CD spectroscopy. Melting temperature (T_m) of DsbA mutants was determined by collecting CD spectra at 222 nm as a function of temperature (40-90 °C). Data were analysed and plotted using GraphPad Prism 8. The results showed that the mutations did not significantly affect thermal stability of the protein, whereby all mutants were more stable in the reduced state relative to the oxidised state.

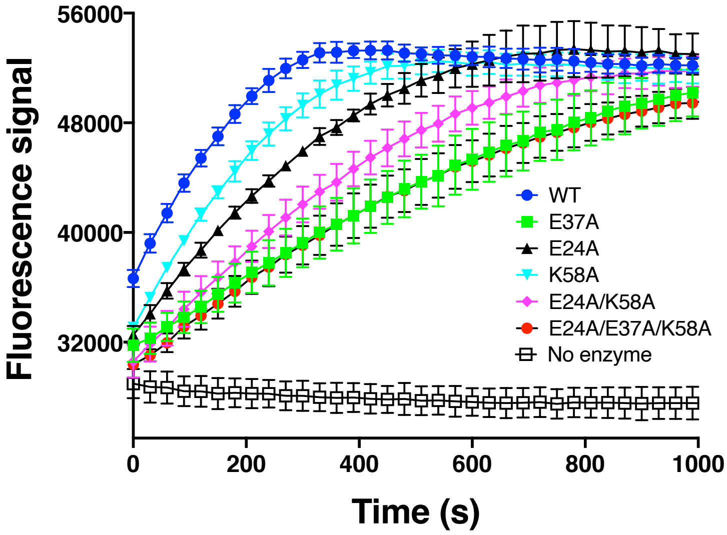

Figure S3. Thiol-disulfide oxidase activity of DsbA mutants was determined by monitoring the oxidation of fluorescently labelled peptide derived from substrate enzyme ASST. Data points shown in the figure are mean ± standard error of means (SEM), n=3.

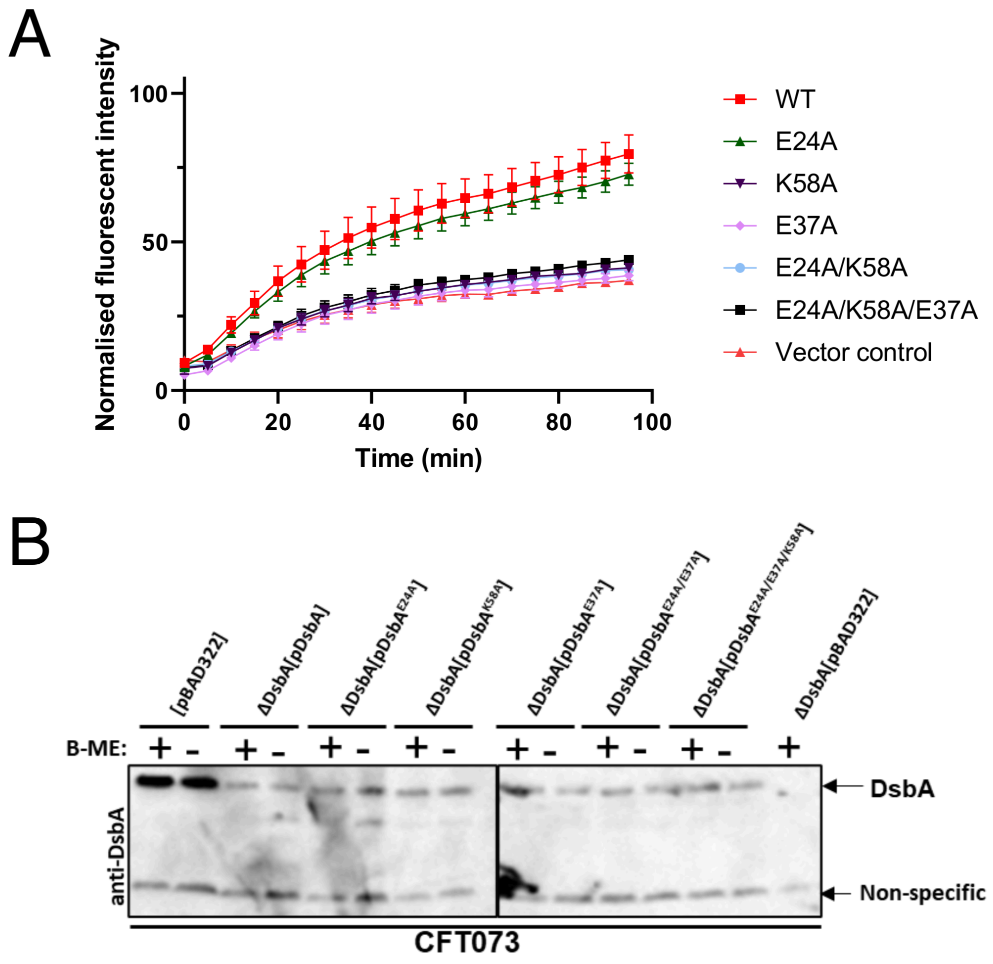

Figure S4. A. Cell-based ASST activity in UPEC CFT073 strains. We investigated the impact of the DsbA mutants *in vivo* using a uropathogenic *E. coli* (UPEC) mutant that was deleted for the *dsbA* gene (CFT073 *ΔdsbA*). Specifically, we monitored the capacity of the mutants to restore the enzymatic activity of plasmid-expressed periplasmic ASST. Overnight cultures of CFT073 *ΔdsbA* [pASST] carrying plasmids expressing wildtype (WT) DsbA and mutants or empty vector control were mixed with phenol (2-10 mM) and MUS (1 mM) and immediately monitored spectrofluorometrically (360/450 nm) for 90 min at room temperature. Sulfotransferase activity by CFT073 [pASST] (defined as 100%) and CFT073 empty vector (defined as 0%) was used to normalise fluorescent intensity readings in each experiment. Data points show mean ± SEM values from six independent experiments. These experiments showed that DsbA K58A and E37A mutations alone or in combination with E24A, but not the E24A mutation alone, in DsbA completely abolished *in vivo* ASST activity, similar to an empty vector control. **B. DsbA protein levels in UPEC CFT073 strains.** We confirmed that protein expression levels were uniform across the complemented strains and were all below chromosomal DsbA protein levels by wildtype CFT073. For that, overnight M9 media cultures of CFT073 and CFT073 *ΔdsbA* carrying plasmids expressing wildtype (WT) DsbA and mutants or empty vector control (pBAD322)

were harvested and lysed in the SDS sample buffer with or without β-mercaptoethanol. Samples were analysed by SDS-PAGE and Western immunoblotting using rabbit polyclonal anti-DsbA sera (1). The ~21 kDa DsbA-specific band is marked at the top by an arrow, alongside a lower molecular weight non-specific band shown as a loading control. We confirmed that protein expression levels were uniform across the complemented strains and were all below chromosomal DsbA protein levels by wildtype CFT073.

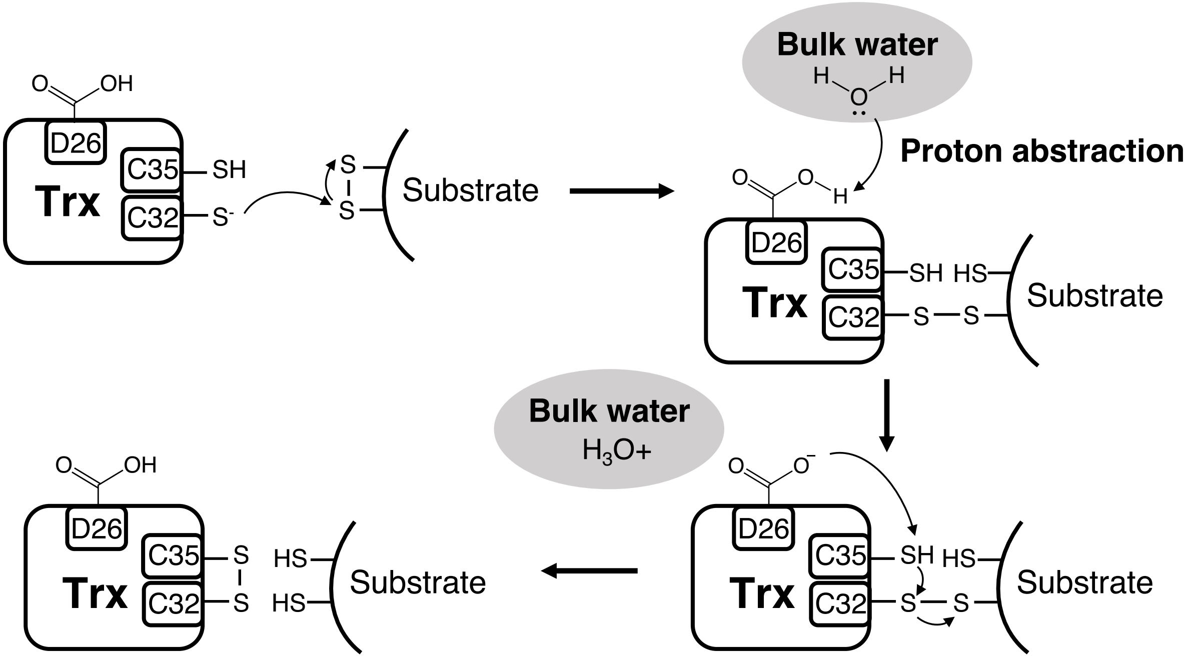

Figure S5. Mechanism of catalysis by reductase thioredoxin proposed by Chivers, P.T. *et al.* It has been proposed by Chivers *et al.* that D26 loses a proton to bulk solvent and then abstracts a proton from C35 to form a C35 thiolate which attacks the nucleophilic C32 of the Trx-substrate mixed-disulfide. This then releases reduced substrate and oxidised Trx. The Trx charged residue/water-mediated deprotonation of C35 would likely minimise futile redox cycling (attack of the mixed disulfide by the other substrate cysteine).

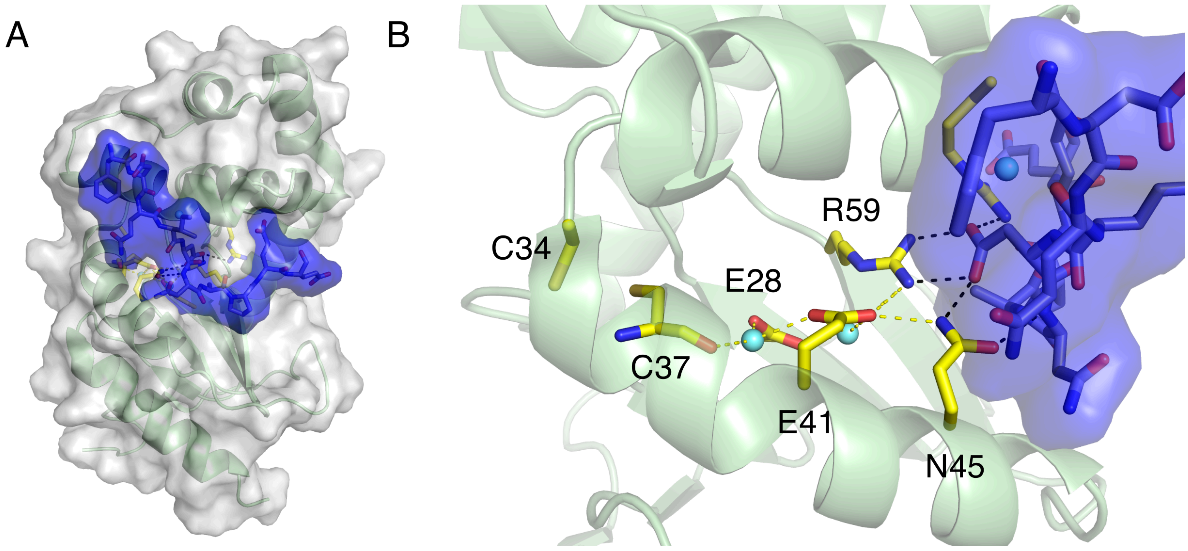

Figure S6. EfTu binds to a non-catalytic charged groove and inhibits AbDsbA oxidase activity. A. Crystal structure of the AbDsbA-EfTu peptide complex (PDB ID:4P3Y). AbDsbA is shown in a green cartoon model with grey surface. EfTu peptide is shaded in blue. B. Expanded view of the AbDsbA-EfTu interaction. The active site cysteines (C34, C37) and polar residues E28, E41, R59 (equivalent residues of EcDsbA E24, E37 and K58) and N45 are shown in yellow sticks. Hydrogen bonds are indicated by yellow/black dashed lines. The EF-Tu peptide (AFDQIDNAPEE) binds AbDsbA tightly, with affinities ranging from 74 to 162 nM. The diothiol oxidase activity of AbDsbA reduced by ~2/3 in the presence of the EF-Tu peptide (5).

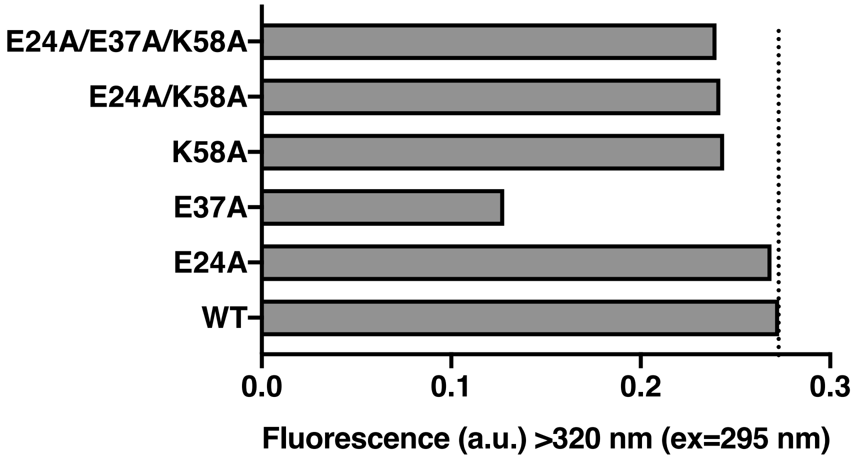

Figure S7. Difference of intrinsic fluorescence of reduced and oxidised DsbA mutants. Intrinsic fluorescence of DsbA variants was monitored with an excitation wavelength of 295 nm and an emission wavelength of >320 nm.

Table S1. List of DsbA crystal structures used for consensus water analysis

		Consensus water			
	PDB ID	w1	w2	w3	w4
1	1FVK	Y	Y	Y	N
2	5QNY	Y	Y	Y	Y
3	5QNZ	Y	Y	Y	Y
4	5QO8	Y	Y	Y	Y
5	5QNW	Y	Y	N	Y
6	5QO9	Y	Y	Y	Y
7	5QNX	Y	Y	Y	N
8	5QO6	Y	Y	Y	N
9	5QNU	Y	Y	Y	Y
10	5QO7	Y	Y	Y	Y
11	5QNV	Y	Y	Y	Y
12	5QO4	Y	Y	Y	N
13	5QNS	Y	N	Y	N
14	5QO5	Y	Y	Y	N
15	5QOC	Y	Y	Y	N
16	5QOA	Y	Y	Y	N
17	5QOF	N	N	Y	N
18	5QOG	Y	Y	Y	N
19	5QOD	N	Y	Y	N
20	5QOE	Y	N	Y	N
21	5QNJ	Y	Y	Y	Y
22	5QO2	Y	Y	N	N
23	5QNQ	Y	Y	Y	Y
24	5QO3	Y	Y	Y	Y
25	5QO0	Y	Y	Y	Y
26	5QO1	Y	Y	Y	N
27	5QNP	Y	N	Y	Y
28	5QNM	Y	Y	Y	N
29	5QNN	Y	Y	Y	N
30	5QNK	Y	Y	Y	N
31	5QNL	Y	N	N	N
32	5QNR	No water modelled in the buried channel			
33	5QNO				
34	5QNI				
35	5QNT				
36	5QOB				

Y= water is present, N= water is not present

Table S2. Data collection and refinement statistics

	**E24A**	**E37A**	**K58A**	**E24A/K58A**	**E24A/E37A/K58 A**
PDB ID	8EQR	8EQQ	8EQO	8EOC	8EQP
Wavelength	0.9537	0.9537	0.9537	0.9537	0.9537
Resolution range (Å)	42.14 - 2.29 (2.372 - 2.29)	49.10 - 2.13 (2.206 - 2.13)	34.18 - 1.623 (1.681 - 1.623)	34.32 - 1.47 (1.523 - 1.47)	61.14 - 2.30 (2.382 - 2.3)
Space group	P 21 21 21	P 1 21 1	C 1 2 1	C 1 2 1	P 1 21 1
Cell dimensions (Å) (a, b, c)	80.83, 108.66, 118.44	61.84, 81.07, 105.247	117.63, 63.09, 74.41	118.01, 63.59, 74.72	61.26, 80.22, 105.73
Angles (°)(α, β, γ)	90, 90, 90	90, 93.64, 90	90 126.651 90	90 126.773 90	90 93.5463 90
Total reflections	328785 (30716)	224091 (21237)	203215 (19836)	279347 (27775)	169359 (16748)
Unique reflections	47306 (4349)	58055 (5758)	54572 (5180)	74558 (7321)	45588 (4521)
Multiplicity	7.0 (6.6)	3.9 (3.7)	3.7 (3.8)	3.7 (3.8)	3.7 (3.7)
Completeness (%)	98.18 (92.32)	99.77 (99.83)	98.69 (94.10)	98.94 (97.51)	99.89 (99.87)
$\langle I/\sigma_I \rangle$	6.10 (1.70)	10.80 (1.62)	20.85 (2.14)	11.65 (1.15)	8.20 (2.65)
Wilson B-factor	29.92	34.29	22.16	23.52	25.73
R_{merge}	0.2131 (0.8738)	0.08022 (0.7303)	0.03312 (0.2234)	0.04748 (0.6711)	0.1078 (0.3692)
R_{meas}	0.2319 (0.9484)	0.09301 (0.8551)	0.03858 (0.259)	0.05525 (0.7803)	0.126 (0.4339)
R_{pim}	0.08926 (0.3614)	0.04639 (0.4389)	0.01951 (0.1293)	0.02787 (0.3936)	0.06446 (0.2244)
$CC_{1/2}$	0.976 (0.828)	0.998 (0.636)	0.999 (0.946)	0.998 (0.728)	0.989 (0.909)
CC*	0.994 (0.952)	0.999 (0.882)	1 (0.986)	1 (0.918)	0.997 (0.976)
Reflections used in refinement	46770 (4338)	58030 (5756)	54570 (5180)	74542 (7316)	45561 (4518)
Reflections used for R_{free}	2535 (255)	2799 (290)	2738 (269)	3781 (386)	2346 (230)
R_{work}	0.2167 (0.2797)	0.1843 (0.3007)	0.1549 (0.1823)	0.1762 (0.2741)	0.1961(0.2352)
R_{free}	0.2588 (0.3249)	0.2308 (0.3336)	0.1838 (0.2075)	0.1967 (0.3082)	0.2534(0.2870)
CC(work)	0.924 (0.876)	0.959 (0.821)	0.971 (0.915)	0.966 (0.831)	0.940 (0.907)
CC(free)	0.898 (0.719)	0.947 (0.716)	0.966 (0.894)	0.968 (0.796)	0.882 (0.835)
Number of non-hydrogen atoms	6460	6427	3449	3314	6344
macromolecules	5885	5932	2971	2948	5882
ligands	34	36	29	29	92
solvent	561	469	465	353	412
Protein residues	752	752	376	376	752
RMS(bonds)	0.008	0.009	0.015	0.006	0.008
RMS(angles)	1.00	1.04	1.26	0.77	0.97

Ramachandran favored (%)	97.04	97.45	98.66	98.39	97.72
Ramachandran allowed (%)	2.96	2.55	1.34	1.61	2.28
Ramachandran outliers (%)	0.00	0.00	0.00	0.00	0.00
Rotamer outliers (%)	1.29	1.27	0.63	0.32	0.64
Clash score	7.12	5.00	3.22	3.58	6.64
Average B-factor	37.00	45.44	28.18	33.24	33.85
macromolecules	36.60	45.39	26.51	32.42	33.71
ligands	43.19	55.63	49.62	56.47	42.39
solvent	41.13	45.48	38.27	39.24	34.75
MolProbity score	1.46	1.40	1.16	1.02	1.24

Table S3. Root Mean Square Deviation (RMSD) of overlaid crystal structures of DsbA wildtype and mutants.

Alignment against crystal structure of oxidised DsbA wildtype (PDB ID: 1FVK)		
	RMSD (Å)	
EcDsbA K58A	0.41	168 atoms
EcDsbA E24A	0.39	171 atoms
EcDsbA E24A/K58A	0.34	168 atoms
EcDsbA E37A	0.49	179 atoms
EcDsbA E24A/E37A/K58A	0.53	174 atoms

Table S4. Strains and plasmids used in this study

**Strains**		
Lab ID	Description	Source
MT542	Wild-type UPEC CFT073	(2)
MT2275	CFT073[pBAD322G]	This study
MT214	CFT073Δ*dsbA*	(1)
MT2280	CFT073Δ*dsbA*[pBAD322G::EcDsbA^{WT}]	This study
MT2281	CFT073Δ*dsbA*[pBAD322G::EcDsbA^{E24A}]	This study
MT2282	CFT073Δ*dsbA*[pBAD322G::EcDsbA^{K58A}]	This study
MT2283	CFT073Δ*dsbA*[pBAD322G::EcDsbA^{E37A}]	This study
MT2284	CFT073Δ*dsbA*[pBAD322G::EcDsbA$^{E24A/E37A}$]	This study
MT2285	CFT073Δ*dsbA*[pBAD322G::EcDsbA$^{E24A/E37A/K58A}$]	This study
MT2286	CFT073Δ*dsbA*[pBAD322G]	This study
MT1600	CFT073Δ*dsbA*[pASST]	This study

MT2501	CFT073Δ*dsbA*[pASST] [pBAD322G::EcDsbA^{WT}]	This study
MT2502	CFT073Δ*dsbA*[pASST] [pBAD322G::EcDsbA^{E24A}]	This study
MT2503	CFT073Δ*dsbA*[pASST] [pBAD322G::EcDsbA^{K58A}]	This study
MT2504	CFT073Δ*dsbA*[pASST] [pBAD322G::EcDsbA^{E37A}]	This study
MT2505	CFT073Δ*dsbA*[pASST] [pBAD322G::EcDsbA$^{E24A/E37A}$]	This study
MT2506	CFT073Δ*dsbA*[pASST][pBAD322G::EcDsbA$^{E24A/E37A/K58A}$]	This study
MT2507	CFT073Δ*dsbA*[pASST] [pBAD322G::EcDsbA^{C33A}]	This study
MT2508	CFT073Δ*dsbA*[pASST] [pBAD322G]	This study
MT1609	CFT073[pSU2718]	(3)
MT1403	CFT073[pASST]	(3)
**Plasmids**		
pASST	pSU2718::AssT	(4)

Supplementary references

1. Totsika, M., Heras, B., Wurpel, D. J., and Schembri, M. A. (2009) Characterization of two homologous disulfide bond systems involved in virulence factor biogenesis in uropathogenic Escherichia coli CFT073. *J. Bacteriol* **191**, 3901-3908

2. Mobley, H. L. *et al.* Pyelonephritogenic Escherichia coli and killing of cultured human renal proximal tubular epithelial cells: role of hemolysin in some strains. *Infect. Immun.* **58**, 1281-1289, doi:10.1128/iai.58.5.1281-1289.1990 (1990).

3. Verderosa, AD., Dhouib, R., Hong, Y., Anderson, TK., Heras, B., Totsika, M. A high-throughput cell-based assay pipeline for the preclinical development of bacterial DsbA inhibitors as antivirulence therapeutics. *Sci. Rep.* 2021;11(1):1569

4. Heras, B., Totsika, M., Jarrott, R., Shouldice, S.R., Guncar, G., Achard, M.E.S., Wells, T.J., Argente, M.P., McEwan, A.G., Schembri, M.A. Structural and functional characterization of three DsbA paralogues from *Salmonella enterica serovar Typhimurium*. *J. Biol. Chem.* 2010; 285:18423–18432.

5. Premkumar, L., Kurth, F., Duprez, W., Groftehauge, M. K., King, G. J., Halili, M. A., Heras, B., and Martin, J. L. (2014) Structure of the *Acinetobacter baumannii* dithiol oxidase DsbA bound to elongation factor EF-Tu reveals a novel protein interaction site. *J. Biol. Chem.* 289, 19869-19880
